# Supplementary material for: A Role for the Proteasome Alpha2 Subunit N-Tail in Substrate Processing
Source: Biomolecules. 2023 Mar 5;13(3):480. doi: 10.3390/biom13030480 (PMC10046698; doi:10.3390/biom13030480)
Supplement: Supplementary file 1 [file biomolecules-13-00480-s001.zip › biomolecules-2200349-supplementary.pdf]

Supplementary Information

A Role for the Proteasome Alpha2 Subunit N-Tail in Substrate Processing

Indrajit Sahu [1,*,†], Monika Bajorek [2,†], Tan Xiaolin [3], Madabhushi Srividya [3], Daria Krutauz [2], Noa Reis [2], Pawel A. Osmulski [3,*], Maria E. Gaczynska [3,*] and Michael H. Glickman [2,*]

[1] Department of Cancer Biology, Dana-Farber Cancer Institute, Harvard Medical School, Boston, MA 02115, USA
[2] Faculty of Biology, Technion—Israel Institute of Technology, Haifa 3525433, Israel
[3] Department of Molecular Medicine, University of Texas Health Science Center at San Antonio, San Antonio, TX 78229, USA
* Correspondence: indrajit_sahu@hms.harvard.edu (I.S.); osmulski@uthscsa.edu (P.A.O.); gaczynska@uthscsa.edu (M.E.G.); glickman@technion.ac.il (M.H.G.)
† These authors contributed equally to this work.

This file contains **Supplementary Figures S1-S5** on the following pages 2-6:

Figures:

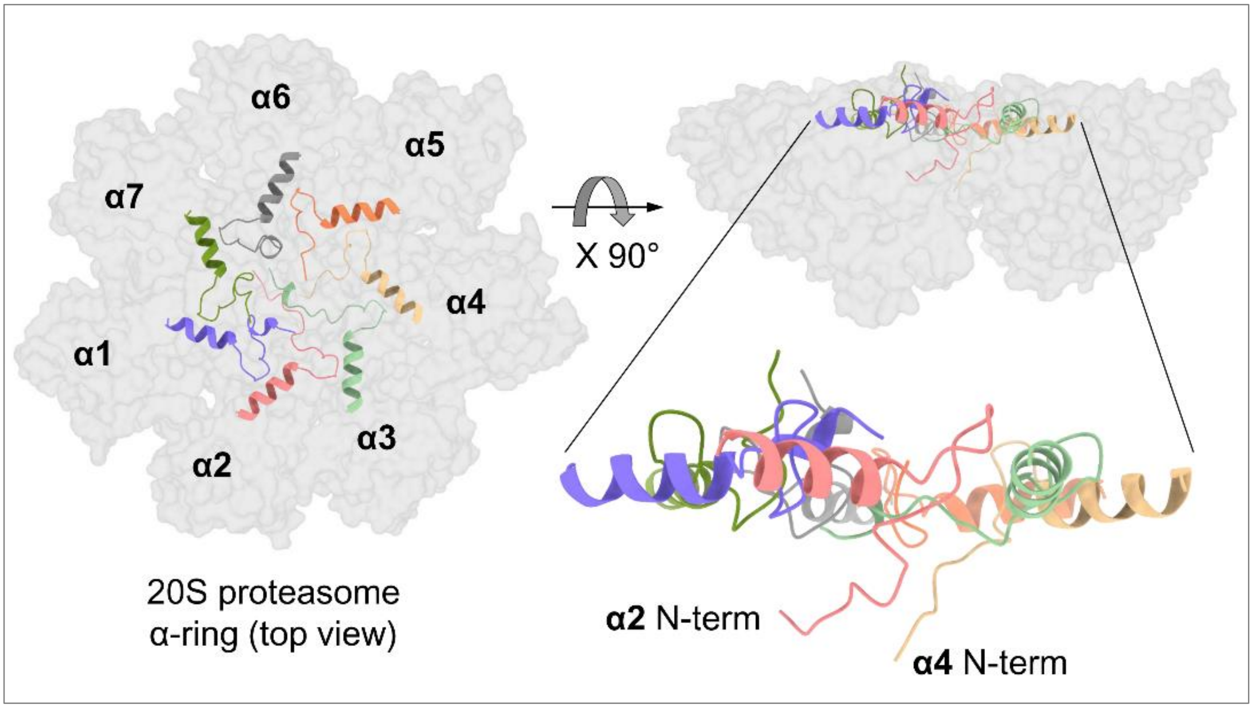

Supplementary Figure S1: The α-ring of closed 20S gate. Position of α-subunit N-termini in closed yeast 20S proteasomes (*Model PDB 1ryp).* Left: Top view of 20S-CP α-ring structure showing in grey color surface contour. The N-terminal tails and the H0-helix of all a-subunits (α N-ring) are shown in colored cartoon. Right: The side view of the figure on left showing the downward position of α2 and α4 N-terminal tails.

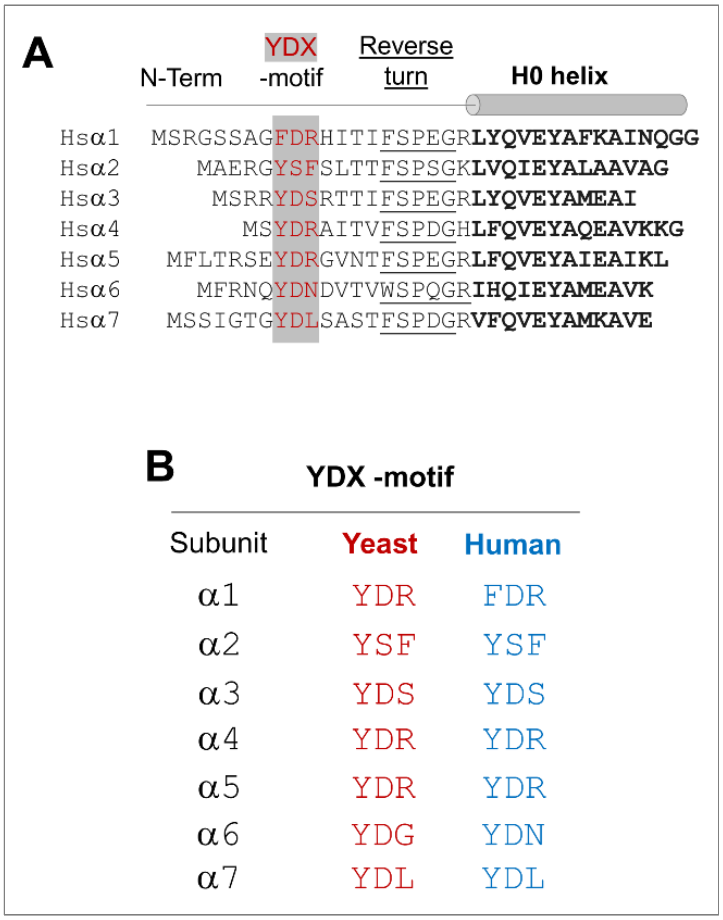

Supplementary Figure S2: YD(X) motifs of α-subunit N-termini of human proteasome. (**A**) N-terminal tail sequences of human proteasome α-subunits highlighting the embedded YD(X) motif. (**B**) Conservation of YD(X) motifs between yeast and human proteasome α subunits.

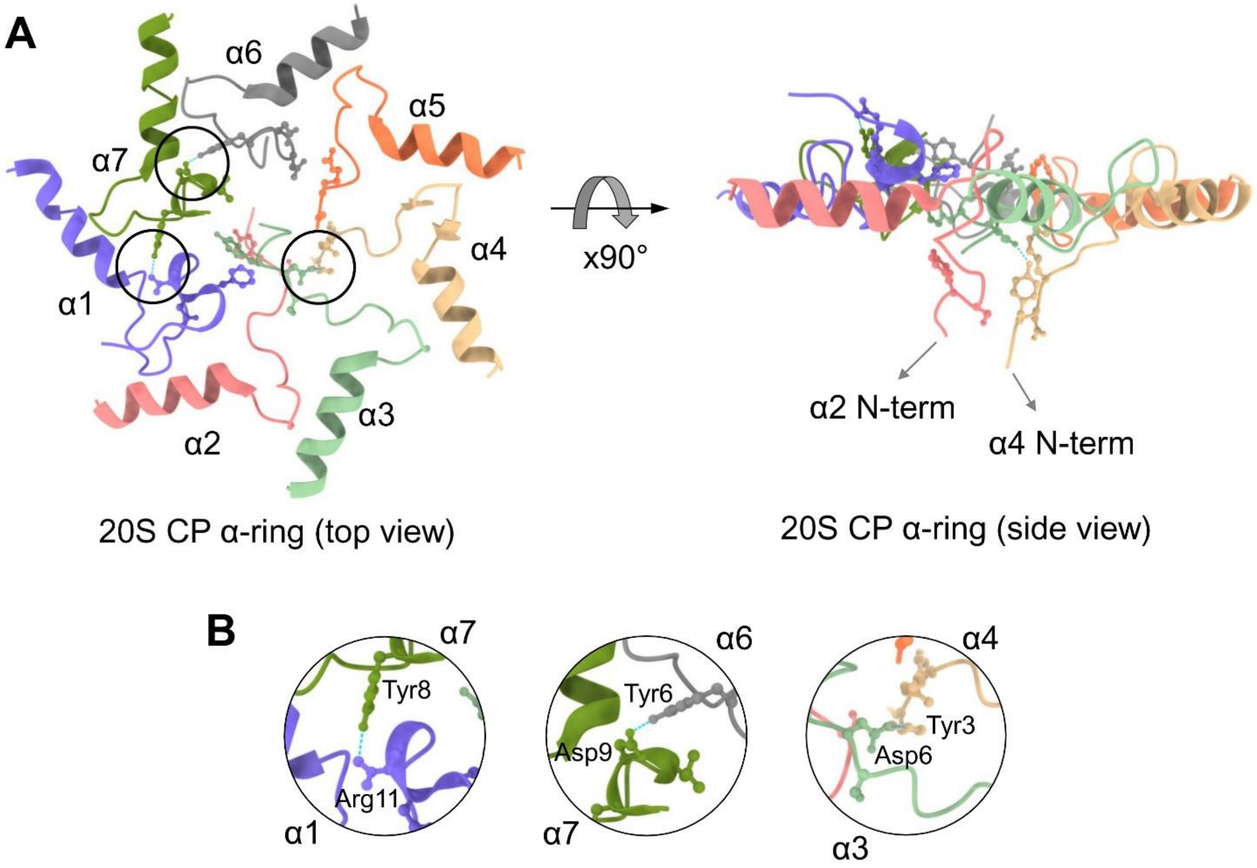

Supplementary Figure S3: YD(X) motif Interactions in closed state human 20S proteasome. (**A**) Left: A model figure of the α N-ring region in resting human 20S (pdb:7pg9) with cartoon representation. The black circle shows the hydrogen bonds between α1-α7, α3-α4 and α6-α7 at the YDX motifs. Right: Side view of the α N-ring shows the deeper extension of α2 and α4 N-termini. The YDX motif of each α-subunit tails are shown in ball-stick representation. (**B**) The zoomed in view of the hydrogen bonds between α1D – Yα7, α3D – Yα4 and α6Y – Dα7.

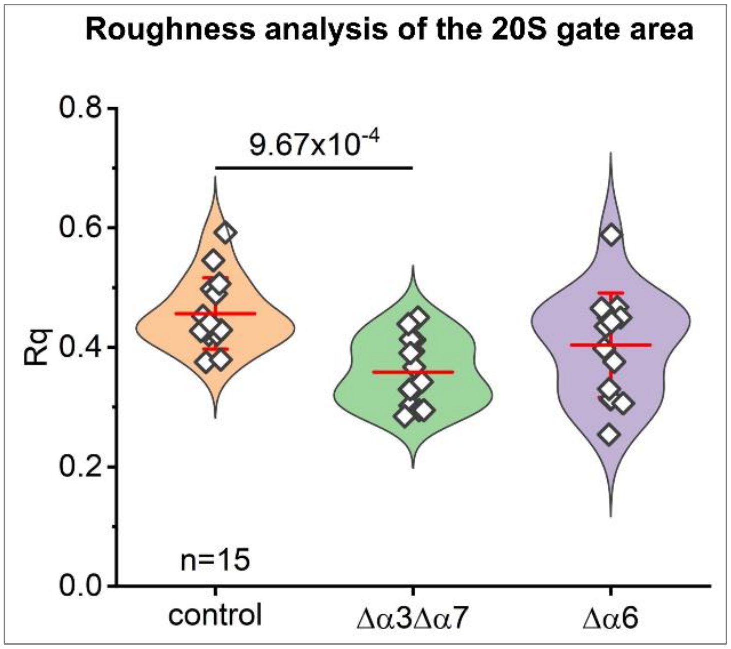

Supplementary Figure S4: Roughness analysis of the 20S gate area. Roughness analysis was performed on the eight-pixel α face scan-lines of randomly selected single images of core particles, fifteen each from WT (control; closed), α3α7ΔN (pseudo-open), and α6ΔN (closed) (NanoScope Analysis and SPIP/Scanning Probe Image Processor v. 6.013; Image Metrology, Denmark). The RMS (root mean square) values were considered a measure of the flatness of the gate area.

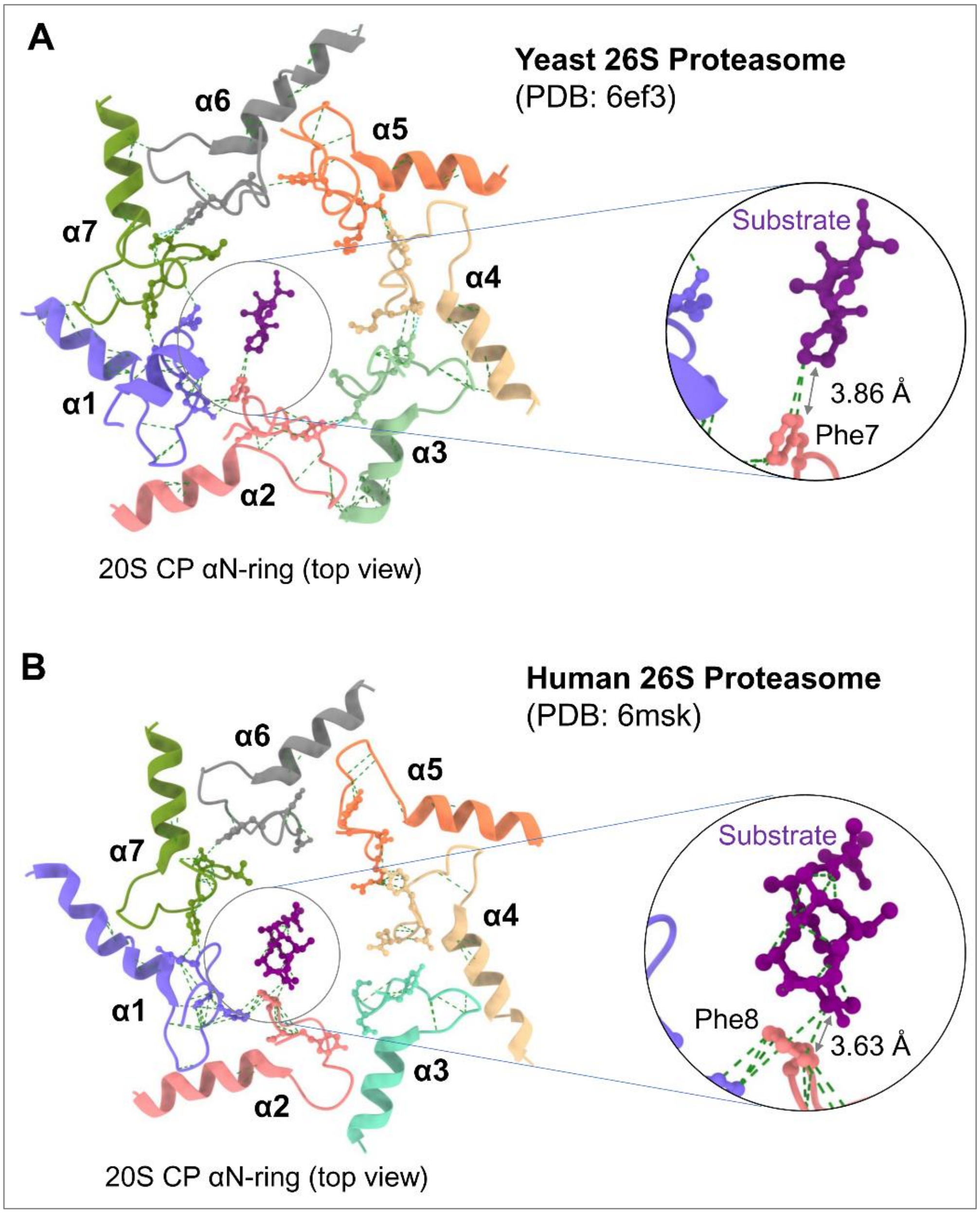

Supplementary Figure S5: Substrate interaction with Phe residue in N-terminal tail of the α2 subunit. Cartoon view models of the α N-ring of Yeast proteasome (**A**) and Human proteasome (**B**) show a substrate polypeptide (in purple ball & stick model) interacting with the Phe residue (in pink ball & stick model) of the corresponding YDF sequence of α2 subunits. Each YD(X) motif of the different α subunits is shown in ball & stick models. The green dash lines represent the Van-der-Wall interactions between atoms within specific residues measured by Chimera X with interaction parameters as "Find pairs of atoms with VDW overlap" ≥ 0.30 Å. The blue dash lines represent the H-bonds between the Y and D residues of adjacent YD(X) motifs.
